# Increased fruit production in *Cipocereus minensis* (Cactaceae) associated with termite nests (Isoptera: Termitidae) in Campo Rupestre (Brazilian altitude grassland)

**DOI:** 10.1371/journal.pone.0335162

**Published:** 2025-11-14

**Authors:** Julya Pires Souza, Laura Simões de Ávila, Tiago Fernandes Carrijo, Carlos Victor Mendonça-Filho, Thiago Santos

**Affiliations:** 1 Graduate Program of Animal Biology, Federal University of Jequitinhonha and Mucuri Valleys, Diamantina, Minas Gerais, Brazil; 2 Graduate Program of Evolution and Diversity, Federal University of ABC, Santo André, São Paulo, Brazil; Universidade de São paulo, BRAZIL

## Abstract

*Cipocereus minensis* (Cactaceae) is a columnar, shrubby cactus endemic to the Campo Rupestre (Brazilian altitude grassland), often found associated with termite nests (Blattaria: Isoptera). This study investigates, for the first time, the association between cacti and termites, exploring the potential influence of termite nests on cactus production and phenology. Specifically, we assessed whether cacti in termite nests exhibited (i) different phenological pattern; (ii) greater reproductive structures produced; (iii) higher buds to immature fruits conversion rate; (iv) different responses in productivity related to temperature and rainfall; and (v) the termite species inhabiting the nests. Weekly quantitative phenological monitoring was conducted on 62 cacti (31 growing on termite nests, and 31 on rocky substrate) over 94 weeks (2018–2020). Temperature and rainfall were measured, and termites identified. Circular statistics were used to assess phenology, while GLMMs tested the effects of temperature, rainfall and substrate on the production of reproductive structures. For both substrates, *C. minensis* flowered and fruited throughout the year, with two to three more pronounced peaks annually, mainly in the dry season. GLMMs indicated that substrate influenced how cacti responded to climate fluctuations, particularly temperature. Seven termite species were identified in 24 inhabited nests, with up to three species per nest, while seven nests were abandoned. Cacti associated with termite nests exhibited greater flower buds and fruit production, and were more affected by climate at the onset of the phenological cycle (*timing*). Termite nests may provide microclimatic regulation (temperature e moisture) and can promote nutrient cycling, acting as “fertility islands” in the nutrient-poor Campo Rupestre soil, suggesting that the cacti grown in this substrate have access to more resources, such as nutrients and moisture.

## Introduction

The Campo Rupestre is a high-altitude Brazilian grassland ecosystem, that sustains a diverse, and endemic biological community, despite covering less than 1% of the Brazilian territory [1–3]. Occurring above 900 m a.s.l., in an extremely old mountaintop ecosystem [4], these rupestrian grasslands are characterized by rocky outcrops [5], a mosaic of vegetation types, a seasonally marked climate, and nutritionally poor, shallow soils with low water retention [4,6,7]. While an estimated 5000 vascular plant species (~2000 endemics) occur in the Campo Rupestre [4,8], knowledge of the fauna and their interactions with plant communities remains surprisingly limited. This study focuses on one such interaction: the association between cacti and termites, two important components of the Campo Rupestre ecosystem.

*Cipocereus minensis*, a columnar, shrubby cactus endemic to the Campo Rupestre of the Meridional Espinhaço Mountain Range, plays a significant ecological and cultural role. Its fruits are a nutritious food source for various species, including humans [9]. Previous studies suggested *C. minensis* exhibited seasonal/subannual flowering and fruiting, with peaks in the dry and rainy seasons, respectively [9,10]. However, recent long-term monitoring has revealed a more complex and unpredictable reproductive phenology, characterized by irregular flowering duration, amplitude, and timing, with flowering pulses lasting from 1 to over 5 months, often concentrated in dry months [11]. This highlights the need to better understand the factors influencing the reproductive success of *C. minensis*.

Phenological studies, which examine the timing, duration, and intensity of plant reproductive events like flowering and fruiting [12], are crucial for deciphering the ecological mechanisms that regulate plant life histories and community dynamics [12–14]. While seasonal climatic patterns (e.g., wet/dry cycles) and competition for pollinators/dispersers are known to influence flowering [12,15,16], the complex interplay of multiple environmental variables (temperature, humidity, soil properties, topography, and biotic interactions) remains poorly understood, particularly in highly variable arid environments [15,17].

Termites (Blattaria: Isoptera) are eusocial cockroaches [18,19] widely recognized as ecosystem engineers [20,21]. They play critical roles in decomposition, soil nutrient cycling, soil aeration, and the creation of “fertility islands,” especially in tropical ecosystems [20,21]. Dominating the soil macrofauna biomass (40–65% in some areas), termites can decompose a significant portion (54–68%) of the total organic matter in certain biotopes [22,23].

Positive interactions between termites and plants are common, yet their ecological significance is not fully understood. While some termites, like *Constrictotermes cyphergaster* and *Nasutitermes acajutlae*, benefit from access to food and water provided by plants [24–26], some plants also benefit from associating with termites. For example, orchids, bromeliads, and *Paepalanthus* species often grow in association with termite nests and foraging galleries [27–29], potentially gaining access to nutrient-rich substrates [20]. However, despite these observations, detailed investigations into these relationships are scarce. To date, only one study has reported termite association with *Paepalanthus bromelioides*(Eriocaulaceae) in Serra do Cipó – MG, but the nature of this interaction was not examined in detail [29].

*Cipocereus minensis* (Cactaceae) growing surrounded by termite nests have been observed in Campo Rupestre areas but remains uninvestigated (Carlos Victor Mendonça-Filho’s personal observation). This study is the first investigation of this association, seeking to determine whether and how termite nests influence cacti phenology. Understanding the processes underlying this association is crucial for assessing its eco-evolutionary significance and contribution to the stability and maintenance of this unique environment.

The aim of this study was to evaluate the productivity and phenology of *Cipocereus minensis* associated with termite nests compared to those growing on rocky substrate in a Campo Rupestre area. Specifically, we assessed whether cacti in termite nests exhibited (i) different phenological pattern; (ii) greater reproductive structures produced; (iii) higher buds to immature fruits conversion rate; (iv) different responses in productivity related to temperature and rainfall; and (v) the termite species inhabiting the nests.

## Materials and methods

**Fig 1:**
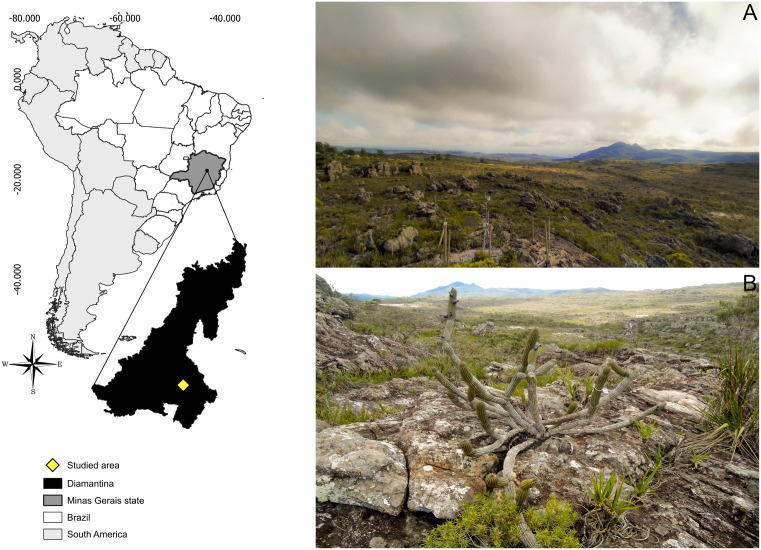
Map of the study area. **(A)** Campo Rupestre area on the Campus JK of the Federal University of Jequitinhonha and Mucuri Valleys (UFVJM), in Minas Gerais state, Brazil. **(B)** A representative picture of the study area and *Cipocereus minensis*. Reprinted from Julya Pires Souza and Laura Simões de Ávila under a CC BY license, with permission from Julya Pires Souza and Laura Simões de Ávila, original copyright 2025.

The study was carried out in a Campo Rupestre area at 1300 m altitude, located in the Meridional Espinhaço Mountain Range, a UNESCO Biosphere Reserve. The site is within the Campus JK of the Federal University of Jequitinhonha and Mucuri Valleys (UFVJM) (18°14’ S, 43°36’ W), in Minas Gerais state, Brazil (Figs 1A and 1B). The regional climate is seasonal, with cold and dry winters (April to September) and hot and wet summers (October to March), while annual rainfall range is from 1250 mm to 1450 mm and mean temperature is 18.5 ºC [30,31].

**Fig 2:**
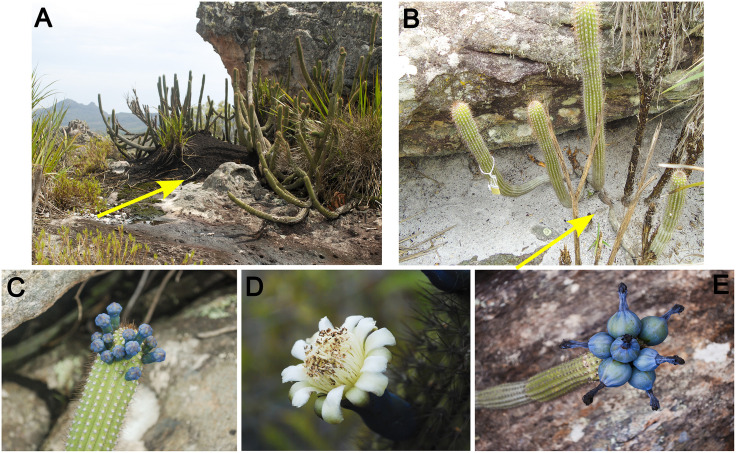
Representation of the individuals on each substrate and the cacti’s reproductive structures. **(A)** Individual with base and roots surrounded by a termite nest; **(B)** Individual on the rock soil; **(C)** Flower buds; **(D)** Flower in anthesis; **(E)** Immature fruits. Yellow arrows indicate the substrate. Reprinted from Julya Pires Souza and Laura Simões de Ávila under a CC BY license, with permission from Julya Pires Souza and Laura Simões de Ávila, original copyright 2025.

To assess the effect of termite nests on the phenology and productivity of *C. minensis*, weekly quantitative phenological monitoring was carried out on 62 cacti, 31 cacti growing on termite nests (Fig 2A) and 31 growing on rocky substrate (Fig 2B), between 2018 and 2020, totaling 94 weeks of observation. The study was divided into study periods, with “Period 1” corresponding from June 2018 to May 2019, and “Period 2” from June 2019 to March 2020. Cacti growing on termite nests were defined as the presence of a nest surrounding most of the cacti’s roots, and in many cases, partially enveloping some branches, regardless of the position of the branches on the nest (on the side, in the middle or on top).

During each observation, the number of flower buds, flowers (anthesis or senescent flowers), and immature fruits present (Figs 2C, 2D and 2E respectively) were quantified [10,32]. Flower anthesis is difficult to register, because it is nocturnal (from around 17:30 pm to 11:00 am), and asynchronous, opening a few flowers per day [9,10]. In addition, five days after anthesis and flower fertilization, the fruit (ovary) begins to develop [10]. Mature fruits could not quantify because they are often predated before reaching full maturity [33].

For the circular analyses, the production of each phenophase of the 31 cacti for each substrate was summed by date, while for Generalized Linear Mixed-effects Models, the data were used separately for each cactus by date.

The number of branches was also quantified for each cactus and used as a covariate in Generalized Linear Mixed-effects Models. For this, each branch was marked with a numbered tag.

Temperature and rainfall data (S1 Fig) were obtained from the National Institute of Meteorology [31]. Average temperature and total precipitation were calculated for the 30 (monthly) and seven days (weekly) preceding each observation.

To test if phenological peaks differed between substrates, and if the peaks were seasonal and synchronized, circular analyses were performed in the Oriana software version 4.02. In this analysis, the average angle represents the average date that the phenological event occurs with the highest frequency. The frequency of occurrence of each phenological event was converted to angles (360◦ representing 365 days of the year, January 1 being the zero angle) and the average data (angle) was calculated. Finally, Rayleigh test was applied to assess the significance of the angle or the mean date of the phenophases with unimodal distribution and to verify if there was a significant seasonal pattern of the mean angle of each phenophase, using a 95% confidence interval. When the mean angle was significant the pattern was considered seasonal, and this corresponds to the average date of the year around which the phenological events are concentrated. The vector *r* varies from 0 to 1 and indicates the concentration of individuals around the mean date or degree of seasonality of the phenophase [34–36]. The peak magnitude was not analyzed.

To test the effect of climatic variables and substrate on the production of reproductive structures, Generalized Linear Mixed-effects Models (GLMMs) were used, with negative binomial residual distribution, corrected for zero inflation and with order 1 temporal autocorrelation structure. Seven variables were used in these models: 1. Mean monthly temperature; 2. Mean weekly temperature; 3. Monthly rainfall; 4. Weekly rainfall; 5. Temperature and substrate interaction; 6. Rainfall and substrate interaction; and 7. Substrate. Ten models were built, ranging from the simplest with one variable to the most complex with seven variables. They were [variables number in parenthesis]: Complete (1–7); Complete without interaction (1–4, 7); Monthly (1, 3, 5–7); Monthly without interaction (1, 3, 7); Weekly (2, 4–7); Weekly without interaction (2, 4, 7); Climatic monthly (1, 3); Climatic weekly (2, 4); Complete climatic (1–4); Substrate (7) (see more details in S1 Table). Cactus’ size (number of branches) was used as a covariate in this modeling, to remove the effect of the cactus’ size in these analyses. Analyses were carried out on R software version 4.2.0 [37,38].

To identify the termites, soldiers and workers were collected (SISBIO #51677) from all 31 termite nests associated with *C. minensis* and identified at the species level using identification keys and consulting experts [39–41, among others]. Soldiers and workers were sampled from each nest associated with the *C. minensis* on the border of the nests, without causing damage to the nests.

## Results

### Reproductive phenology

**Fig 3:**
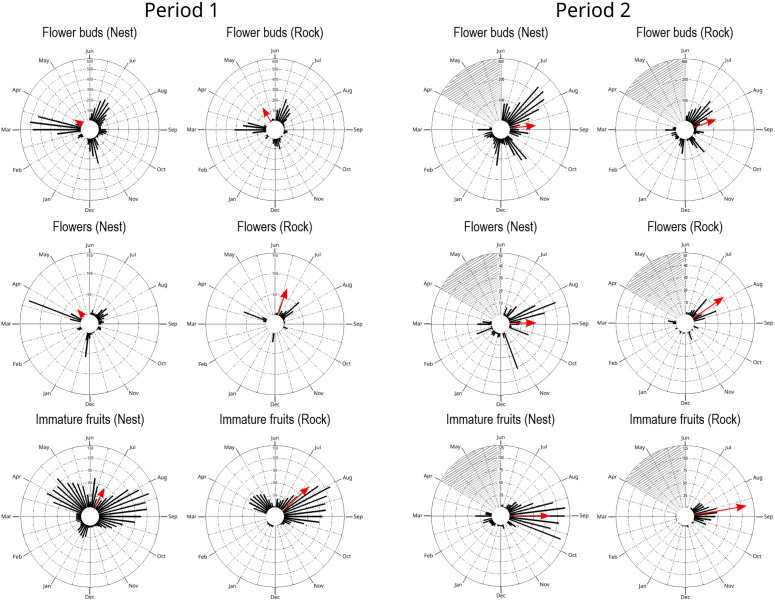
Circular histograms representing the production of flower buds, flowers and immature fruits of *Cipocereus minensis.* The results show the total production of the 31 cacti growing on termite nest and of the 31 cacti growing on rocky substrate, from June 2018 to May 2019 (Period 1), and from June 2019 to March 2020 (Period 2). Red arrows point to the average month of production (mean angle: μ), and the length of the arrow represents the mean length of the vector (r) of each cactus (the degree of seasonality). Grey shaded area represent the months that were not studied in 2020 (April to June). Histograms are not on the same scale.

*Cipocereus minensis* flowered and fruited throughout the year, albeit with oscillations between high and low yields (Fig 3). Generally, the most pronounced flowering and fruiting peak occurred in the dry season, between April and August, for both substrates, however, two to three pronounced peaks were observed annually for each phenophases.

Overlapping phenophases were observed on the same individual, with flower buds, anthesis/senescent flowers, and immature fruits being present simultaneously, regardless of the substrate. Flowering was asynchronous, opening a few flowers per day, making it difficult to monitor and, therefore, the fruits also matured at different moments, resulting in relatively extended fruiting peaks. Generally, the fruits were predated before reaching full maturity, and for this they were not quantified.

During the first period of the study (June 2018 to May 2019), three pronounced flowering peaks were observed (mid-July, mid-November and mid-March) (Fig 3). Cacti growing on termite nests produced more flower buds (e.g., total summing up 31 cacti on 07/03/2019, Nest = 508 buds and Rock = 231 buds), although overall phenology was similar between substrates. While flowering occurred on similar dates for both substrates, it was more concentrated in cacti growing on the rocky substrate (e.g., total summing up 31 cacti: 20 flowers on July 11; 58 on July 17; 33 on July 25; and 6 on August 2). In contrast, flowering in cacti growing on termite nests was distributed more evenly over several weeks, with sustained production from July to mid-August (e.g., total summing up 31 cacti: 24 flowers on July 11; 35 on July 17; 20 on July 25; 29 on August 2; 17 on August 8; 10 on August 15; and 7 on August 21). Fruiting occurred throughout the year on termite nests but was absent on the rocky substrate in November, February, and the first half of March.

The second period (June 2019 to March 2020) presented a continuous flower bud peak, with similar patterns on both substrates (Fig 3). As in the first period, flower production was more concentrated in cacti growing on the rocky substrate and more evenly distributed in cacti growing on termite nests. Fruiting was more pronounced in cacti growing on termite nests from January to March, whereas it was nearly absent in cacti growing on the rocky substrate during this period (e.g., total summing up 31 cacti on 05/02/2020, Nests = 12 immature fruits and Rock = 1 immature fruit; and on 03/03/2020, Nests = 17 immature fruits and Rock = 1 immature fruit).

Nevertheless, circular statistical analysis (Table 1) showed that in Period 1, the mean fruiting date was late June 2018 for cacti growing on termite nests (µ _nests_ = 301.66°), and in the second half of July 2018 for cacti growing on the rocky substrate (µ _rock_ = 330.05°). Flower buds and flowers could not be compared between substrates, because the data did not follow a uniform distribution for the termite nest substrate. In Period 2, the mean flower buds peak date was similar for cacti growing on both substrates, occurring in mid-August (µ _nests_ = 82.89° and µ _rock_ = 71.91°). The mean flowering date was late July for cacti growing on the rocky substrate (µ _rock_ = 56.27°) and late August for cacti growing on termite nest substrate (µ _nests_ = 87.76°). As a result, the fruiting mean date also was later for cacti growing on termite nest substrate (µ _nests_ = 124.03° and µ _rock_ = 80.30°).

**Table 1:** Circular statistic results for phenology and seasonality.

Year	Phenophase	Substrate	Mean vector (µ)	95% Confidence Interval	Length of mean vector (r)	Rayleigh Test (Z)	Rayleigh Test (p)
**Period 1**	Flower buds	Nest	301.66°	--	0.14	97.72	**<0.001**
		Rock	330.05°	324.16° 335.94°	0.26	175.69	**<0.001**
	Flowers	Nest	319.52°	--	0.31	12.90	**<0.001**
		Rock	18.95°	9.14° 28.77°	0.99	58.66	**<0.001**
	Fruits	Nest	27.41°	22.95° 31.88°	0.35	295.56	**<0.001**
		Rock	49.41°	46.17° 52.64°	0.57	488.67	**<0.001**
**Period 2**	Flower buds	Nest	82.89°	79.48° 86.31°	0.41	491.85	**<0.001**
		Rock	71.91°	66.86° 76.96°	0.38	228.47.	**<0.001**
	Flowers	Nest	87.76°	76.62° 98.91°	0.42	46.05	**<0.001**
		Rock	56.27°	45.07° 67.47°	0.59	40.06	**<0.001**
	Fruits	Nest	124.03°	120.38° 127.69°	0.65	351.51	**<0.001**
		Rock	80.30°	77.05° 83.56°	0.85	265.45	**<0.001**

Table 1. Circular statistic results for phenology and seasonality to test if in each phenophase there was a difference in the phenological peaks and if these peaks were seasonal between substrates. This study was carried out with 31 cacti growing on termite nest and 31 cacti growing on rocky substrate, from June 2018 to Mai 2019 (Period 1), and from June 2019 to March 2020 (Period 2), totaling 94 weeks. Nest: termite nest substrate; Rock: rock substrate.

Rayleigh test indicated significant seasonality in all phenophases throughout two years of this study (p < 0.01, Table 1). However, flowers for cacti growing on the rocky substrate in Period 1 (r _rock_ = 0.99) and fruits in both substrates in Period 2 (r _nests_ = 0.65 and r _rock_ = 0.85) exhibited higher degree of seasonality, with most of their production occurring during the dry season (April to September).

### Generalized linear mixed-effects models

The GLMMs analysis indicated that not only cacti growing surrounding by termite nests produce more, but the difference in substrate also influenced how cacti responded to climatic fluctuations, especially temperature. The Complete model best explained flowering yield (buds and flowers), while the Monthly model best explained fruiting yield (see AICc and ΔAICc in S2 Table).

When modeling the effect of substrate alone on bud, flower, and fruit production, it became evident that cacti growing on rocky produced fewer fruits (β = −1.069, p = 0.043) and buds (β = −1.123, p = 0.030) than those growing on termite nests (Table 2). However, when the best models for each phenophase were analyzed, it became clear that flower bud, flower, and fruit production were all significantly constrained by climatic drivers, with consistent negative associations with temperature. Fruit production declined with mean monthly temperature (β = −0.276, p = 0.002). Flower production was also reduced by mean monthly temperature (β = −0.861, p = 0.001), while bud production showed a similar negative association (β = −0.388, p = 0.035). Substrate type alone did not significantly affect reproductive output, but it modulated the influence of climatic variables. For fruits, both accumulated monthly precipitation (β = −0.003, p = 0.001) and mean monthly temperature (β = −0.141, p = 0.003) imposed stronger negative effects on individuals established on rocky substrates compared to those growing on termite nests. For flowers, accumulated monthly precipitation (β = −0.004, p = 0.011) and mean weekly temperature (β = −0.466, p = 0.003) similarly exerted stronger negative effects on plants growing on rocky substrate. For flower buds, the interactions of mean monthly temperature (β = 0.263, p = 0.004), mean weekly temperature (β = −0.246, p = 0.001), and accumulated weekly precipitation (β = −0.004, p = 0.045) highlighted that the negative effects of climate were amplified cacti growing on rocky substrates compared to those growing on termite nests.

**Table 2:** Results of the generalized linear mixed-effects models.

Predictors	Flower buds			Flowers			Fruits		
	β	Standard error	p	β	Standard error	p	β	Standard error	p
Intercept	6.856	1.806	**<0.001**	6.076	2.358	**0.010**	4.169	1.676	**0.013**
Substrate (Rock vs. Nest)	−1.425	0.978	0.145	2.144	1.515	0.157	1.520	0.988	0.124
Mean monthly temperature	−0.388	0.184	**0.035**	−0.861	0.254	**0.001**	−0.276	0.089	**0.002**
Monthly rainfall	0.001	0.001	0.398	0.000	0.002	0.928	−0.002	0.001	0.078
Mean weekly temperature	−0.020	0.152	0.893	0.347	0.208	0.095	--	--	--
Weekly rainfall	−0.002	0.004	0.649	0.007	0.005	0.155	--	--	--
Number of branches	0.023	0.020	0.243	0.008	0.015	0.584	0.003	0.020	0.862
Substrate (Rock vs. Nest) x Monthly rainfall	0.000	0.001	0.559	−0.004	0.002	**0.011**	−0.003	0.001	**0.001**
Substrate (Rock vs. Nest) x Mean monthly temperature	0.263	0.092	**0.004**	0.314	0.182	0.084	−0.141	0.048	**0.003**
Substrate (Rock vs. Nest) x Weekly rainfall	−0.004	0.002	**0.045**	0.005	0.004	0.269	--	--	--
Substrate (Rock vs. Nest) x Mean weekly temperature	−0.246	0.076	**0.001**	−0.466	0.155	**0.003**	--	--	--

Table 2. Results of the Generalized Linear Mixed-effects Models to evaluate the effect of climate and substrate on the production of flower buds, flowers and fruit.

-- indicates that these weekly models were not used, because the model that best explained the results was the Monthly Model (see AIC method in S2 Table).

### Termite identification

**Fig 4:**
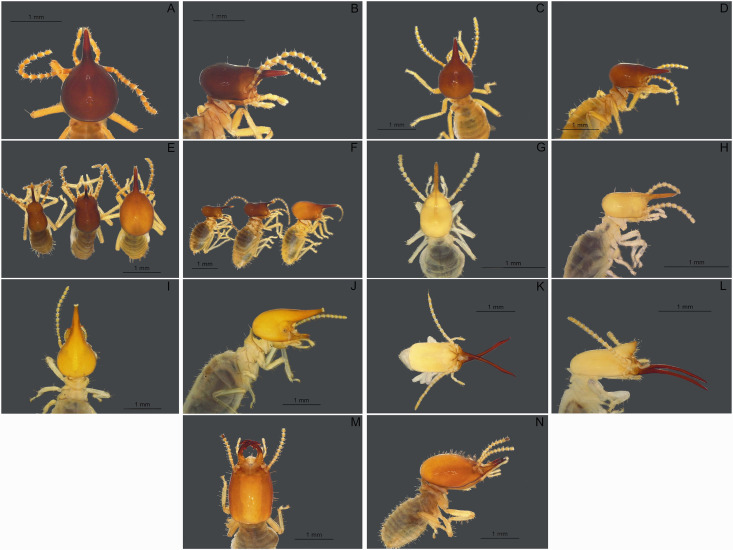
Termite species found in nests surrounding *Cipocereus minensis.* **(A, B)** *Nasutitermes* cf. *coxipoensis*; **(C, D)** *Nasutitermes* sp. 1; **(E, F)** *Diversitermes diversimilis*; **(G, H)** *Subulitermes* cf. *baileyi*; **(I, J)** *Silvestritermes euamignathus*; **(K, L)** *Termes* cf. *fatalis*; **(M, N)** *Amitermes bandeirai*.

Among the 31 nests associated with the cacti, 24 nests were inhabited, while seven nests were abandoned, but showed no signs of deterioration. An average of 1.00 species nest-1 (1.096 ± 0.83) was verified, however, up to three species were found in the same nest. Seven species of termite were identified: *Nasutitermes* cf. *coxipoensis*, *Nasutitermes* sp. 1, *Diversitermes diversimilis*, *Subulitermes* cf. *baileyi* (Nasutitermitinae), *Silvestritermes euamignathus* (Syntermitinae), *Termes* cf. *fatalis* (Termitinae) and *Amitermes bandeirai* (Amitermitinae) (Fig 4). Distinguishing between builders and inquilines was difficult. Furthermore, foraging galleries were observed at the base to two cacti growing on the rocky substrate. In one *Silvestritermes euamignathus* was observed, while *Nasutitermes* cf. *coxipoensis* was in another one.

## Discussion

Cacti associated with termite nests showed greater production of reproductive structures, particularly fruits, compared to those growing on rock substrate. This difference is evident both in average production and in the cacti’s responses to variations of temperature and rainfall. The results suggest that climate variables influence more in the phenology at the onset of the cycle (*timing*), while the substrate, together with the climatic variations, influences the increase of flower and fruit production.

The most pronounced peak in flowering occurred in the dry season, while the largest fruiting peak occurred at the onset of the wet season. This pattern was also encountered in previous phenological studies on *C. minensis* [9–11]. However, unlike these earlier studies, we observed up to three phenological peaks, with inter-annual and irregular variation in duration, amplitude, and timing (months or seasons), regardless of the substrate, as previously reported [11]. While *C. minensis* has been classified as having a subannual flowering pattern (two peaks per year) (see classification in [42]) proposed in another research [9], here we observed a continuous flowering pattern, consistent with Mendonça-Filho et al. [11]. In our case, there are some more pronounced peaks, but also some smaller, constant peaks throughout the year. This continuous pattern strategy also is common in other columnar cacti species, like *Stenocereus griseus, Pilosocereus leucocephalus, Melocactus glaucescens* and *Melocactus albicephalus* [16,43,44].

We observed that *C. minensis* exhibited substantial flower bud loss (due to dropping or failure to develop), as well as instances of unpollinated flowers and fruit abortion. This helps explain why the number of fruits is much lower than the number of flower buds produced. The prevalence of more expressive flowering peaks in the dry season can be advantageous, because in open environments like the Campo Rupestre, thunderstorms with heavy rain and strong winds can damage or drop buds and flowers, especially those in an apical position [45,46].

We constructed several statistical models to test which variables best explain why cacti in termite nests had higher production. The Complete model better explained the flowering yield (buds and flowers), while the Monthly model explained the fruiting yield. Climatic conditions more appropriate for the reproduction of cacti (lower or higher temperatures, and accumulated rainfall) can provide greater reproductive structures production in cacti associated with termite nests. Nevertheless, Mendonça-Filho et al. [11] observed a higher number of flowers in dry and cold months compared to wet and hot months, although statistically flower production was not significantly related to temperature and negatively related to rainfall.

Rojas-Sandoval and Meléndez-Ackerman [47] suggested that the interaction between endogenous developmental and environmental signals (e.g., primary: temperature and rainfall; and secondary: nutrient available in the soil and day length) can determine reproductive activity in columnar cacti. Three main reproductive strategies for buds and flowers production have been documented: i) primarily controlled by rainfall [15,48,49]; ii) primary controlled by temperature [12,16]; and iii) control by multiple factors, such as temperature influencing flower buds, while rainfall influences flower production [47]. Furthermore, soil type and characteristics, climate, geographic location, and other environmental factors contribute to these diverse reproductive strategies.

Our results indicate that, even under unfavorable climatic conditions (e.g., constant climatic oscillation, extreme low and high temperatures, etc.), cacti associated with termite nests appear to benefit from this association. This benefit may be attributed to the microclimatic control system within termite nests, which temperature and moisture regulation, that maintain the termite assemblage survival, but which can impact an entire surrounding community. Different types of microclimatic control have been developed by termite assemblages, depending on the environmental conditions in which they live. As consequence, the differences in the epigeal nest structure are correlated with the ambient temperature. In larger colonies in savannah areas, for example, nests can have a reduced surface area (e.g., the wall can be thinner), and an increased surface complexity, as well as the ability to exchange gases directly with the air or the complex and efficient ventilation system via circulation of air [50–53].

In addition, moisture and nutrient hotspots are formed within nest soil, foraging galleries and their surroundings. In savanna areas, these hotspots patches lead to multiple cascading effects on both vegetation and animal communities, like communities of wood and herbaceous plants found on and around the nests [54,55]. A study in South Africa savanna, for example, showed that the height of the nest varied across the rainfall gradient. Higher nests have longer shadow lengths, altering microclimatic conditions, such as moisture, on different sides of the nests, and consequently favoring grass species adapted to such conditions [54]. Regardless of the specific mechanism promoted by different termite species, in general, temperature and moisture remain stable, providing a more stable thermal range that can benefit cacti during reproductive period.

Regarding nutrient hotspots, studies have shown that termites significantly modify soil properties both vertically and horizontally. In the decomposition of matter process, they ingest organic and inorganic matter, promoting nutrient cycling and can creating “fertility islands” (spots very rich in nutrients), which positively impact vegetations and the surrounding community [20,54–59]. In the nutrient cycling process, termite assemblages mediate the carbon (C), nitrogen (N), phosphorus (P) and potassium (K) cycles in the soil [59]. Also, they provide better fixation of nitrogen and carbon, and increase soil porosity, density, aeration, and water infiltration [20,58,60–62]. Termite nests create nutrient-rich sites for germination and growth [58], exhibiting high concentrations of C, N, clay, and exchangeable cations in and around the nests, providing higher nutrient availability for vegetation [20,50,55,60,61,63,64].

As observed in the fungus-growing termite genus *Macrotermes* (Macrotermitinae), living in the African savannahs, this termite genus redistribute soil particles on scales of tens of meters, ultimately influencing mineral composition, hydrology, drainage, topography, fluxes of nutrients in landscapes, and specially, their large mounds typically contain greater concentrations of macronutrients as N, P, and major cations, as well as micronutrients, such as manganese (Mn), cobalt (Co), cooper (Cu) and selenium (Se) [55,65]. Similar processes in Brazil have already been observed in rain forest areas in the Atlantic Forest and savannah [66,67], and in Ironstone Rupestrian Grassland (Canga) [59]. Sarcinelli et al. [66,67] showed that termite nest, inhabited by both builders and inquiline, presented differences in soil chemical characteristics between termite nests and adjacent soil, especially the amount of organic carbon (OC), which was up to 11.9 times higher, and N. Schaefer et al. [59] showed that termite activity significantly increased the concentration of organic matter, available P and exchangeable calcium (Ca), K, N, magnesium (Mg) in the soil, and these differences were relatively more relevant to grassland vegetation compared with forests.

Campo Rupestre soils are characteristically nutrient poor. Especially in quartzite areas, the soil consists of a fine layer of white sand overlying the rock, that are acidic and aluminum saturated. These soils exhibit very low levels of essential nutrients, including K, Ca, Mg, exchangeable cations, and are particularly deficient in N, P, and water retention [4,6,68]. Thus, open vegetations such as Campo Rupestre and savannahs, which have nutritionally poorer and drier soils, seem to benefit from all the soil cycling and modification processes provided by termites and their nests.

Seven termite species were associated with *C. minensis*, with up to three species occurring in the same nest. Three out of the seven species are known to be nest builders: *S. euamignathus, T. fatalis* and *N. coxipoensis*, each one with its own specific construction pattern [40,69]. Most nests appeared to have been constructed by *S. euamignathus.* However, identifying the builder and inquiline species proved challenging. Two builder species were observed in the same nest more than once (*N.* cf. *coxipoensis* and *T.* cf. *fatalis*; *N.* cf. *coxipoensis* and *S. euamignathus*), but they were collected at different times. It is not common to find inquilines in *Nasutitermes* nests [71, and authors’ personal observation].

Abandoned nests can be recolonized by new species of termites [70], as well as by other organisms such as wasps and other insects, fungi, or even arthropods [70–73], and can continue to act on the dynamics of the ecosystem, nutrient cycling, and the maintenance of biodiversity [70]. In the literature, for example, there are records of *S. euamignathus* remodeling abandoned nest of other species [40], as well as several species of the genus *Termes* behaving as inquilines in the Neotropical region [69]. Furthermore, Mathews [69] had already observed that in Cerrado areas, nests of *S. euamignathus* can have *Nasutitermes kemneri* inhabiting their outer cells, while in Pantanal, *Dentispicotermes pantanalis* (Amitermitinae) and *Anoplotermes* spp. (Apicotermitinae) inhabited the lower part of the nest. The presence of two species such as *N. kemneri* and *S. euamignathus* in the same nest can result in an increase of *N. kemneri* colony and decline of *S. euamignathus* colony, or the aggressive behavior of *N. kemneri*.

In addition to *C. minensis*, other plant species have also been observed growing in association with termite nests in this region, many of which are endemic to Campo Rupestre (e.g., *Pilosocereus aurisetus* (Cactaceae), *Vellozia* spp. (Velloziaceae), Orchidaceae, Bromeliaceae, Asteraceae, Cyperaceae, Malpighiaceae, Melastomataceae, Fabaceae, among others) (authors’ personal observation). However, despite knowledge about the impacts of termite nest on vegetation and soil presented here, it has not yet been possible to clarify what impacts cacti may have on colonies, whether positive or negative. It is also unclear to us at what moment this association is established. Personal observations, apart from this study, have shown both scenarios: very large and developed nests that had cacti beginning to grow, as well as a very large and developed cactus with a small nest connected, through a foraging gallery, to another termite nest next to it. This nest also does not make it clear to us whether it was a secondary nest or just a foraging area for the termites (Julya Pires Souza’s personal observation).

Finally, given the above discussion, our results suggest that cacti growing in association with termite nests have greater available resources, including nutrients and moisture. Further research is needed to explore the properties of both substrates. Also, it is necessary to expand the studies of termites and plant phenology in Campo Rupestre areas, to understand the ecological process that sustains these communities.

## Conclusion

This study demonstrates that individuals of *Cipocereus minensis* growing on termite nests exhibited a significantly higher production of flower buds, flowers and fruits compared to those growing on rocky substrate: the typical nutrient-poor soils of the Campo Rupestre. This enhanced reproductive output strongly suggests that cacti benefit from the presumed higher nutrient and moisture availability within termite nests, resources critical for reproductive success in arid and semiarid environments. Future research is needed to fully elucidate the complex ecological processes, including plant-termite interactions, that sustain the unique and threatened biodiversity of the Campo Rupestre. Preserving this ecosystem is paramount for safeguarding its endemic species and maintaining vital ecosystem services.

## Supporting information

S1 Fig Climatic data. Annual (A) and monthly (B) climate data showing accumulated rainfall and average temperature for Diamantina, Minas Gerais, Brazil. The light gray bar and light gray point indicate incomplete data for this year.
(PNG)

S1 Table Generalized Linear Mixed-effects Models. Structure of the Generalized Linear Mixed-effects Models adjusted to test the best set of variables to explain the production of reproductive structures of *C. minensis* cacti associated or not with termite nests. 1. Mean monthly temperature is the mean temperature of the 30 days prior to collection; 2. Mean weekly temperature is the mean temperature of the seven days prior to collection; 3. Monthly rainfall is the accumulated rainfall in the 30 days prior to collection; 4. Weekly rainfall is the accumulated rainfall in the seven days prior to collection; 5 Temperature and substrate interaction are the possible interactions between weekly and monthly temperature variations and the termite nest and rock substrates; 6. Rainfall and substrate interaction are the possible interactions between the weekly and monthly rainfall variations and the termite nest and rock substrates; 7. Substrate are the two substrates to which the cacti are inserted – termite nest and rock substrate. * This formula indicates that possible differences between the cacti in the rock substrate or termite nest were not considered in terms of excess zeros.* The correction for excess zeros was only applied to the fruit and bud models, as it was not necessary for the flower models.
(PDF)

S2 Table Results of the AICc and ΔAICc method. The AICc and ΔAICc method were used to check which sets were the most parsimonious and best explained the variations. k represents the number of parameters used.
(PDF)

S3 Table Data Availability. All the data collected in this study is available in this table.
(XLSX)
