# Supplementary figures and images for: Increased fruit production in Cipocereus minensis (Cactaceae) associated with termite nests (Isoptera: Termitidae) in Campo Rupestre (Brazilian altitude grassland)
Source: PLoS One. 2025 Nov 14;20(11):e0335162. doi: 10.1371/journal.pone.0335162 (PMC12617911; doi:10.1371/journal.pone.0335162)

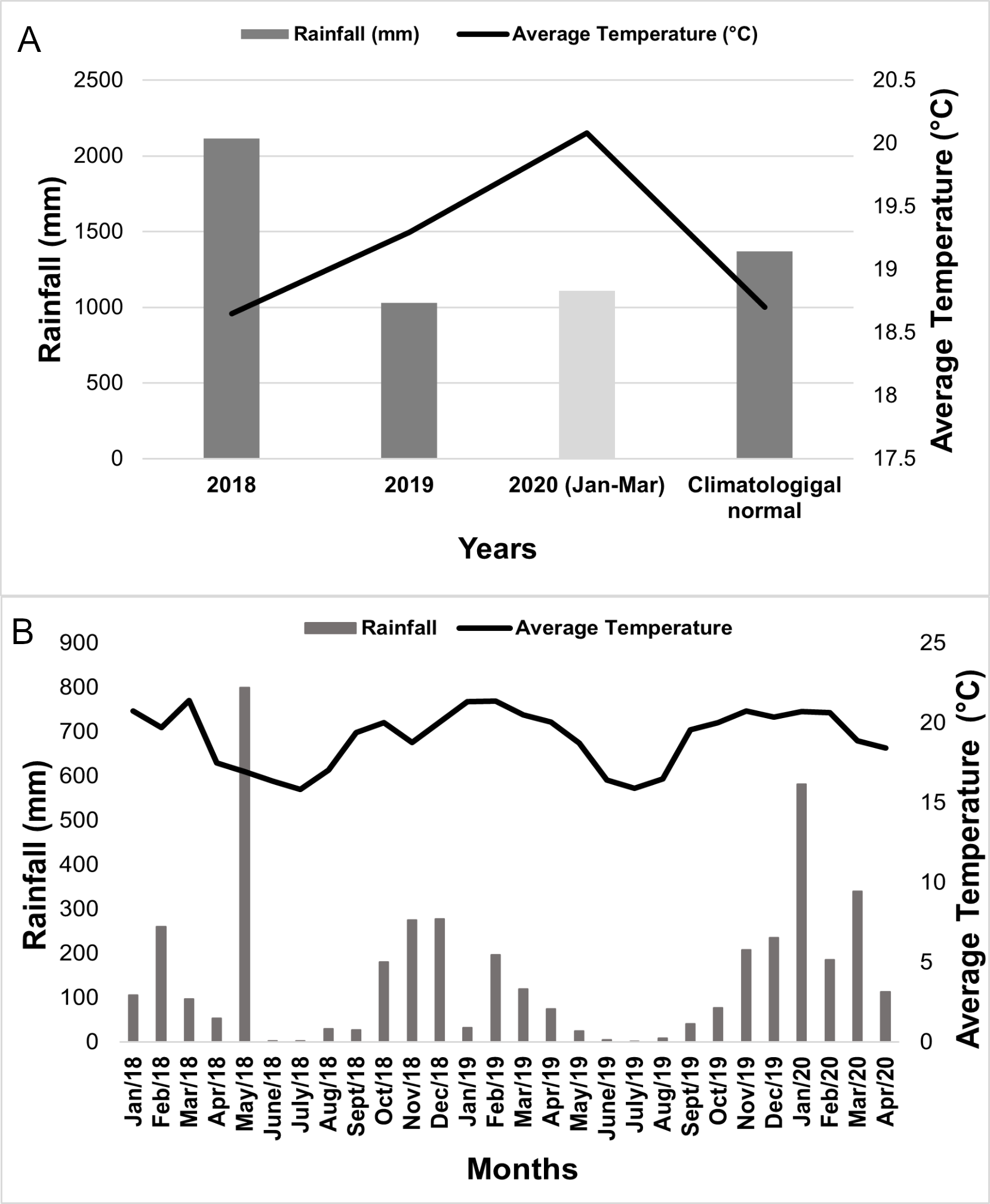

Supplement: S1 Fig — Annual (A) and monthly (B) climate data showing accumulated rainfall and average temperature for Diamantina, Minas Gerais, Brazil. The light gray bar and light gray point indicate incomplete data for this year. (PNG) [file pone.0335162.s001.png]
