# Supplementary material for: Increased fruit production in Cipocereus minensis (Cactaceae) associated with termite nests (Isoptera: Termitidae) in Campo Rupestre (Brazilian altitude grassland)
Source: PLoS One. 2025 Nov 14;20(11):e0335162. doi: 10.1371/journal.pone.0335162 (PMC12617911; doi:10.1371/journal.pone.0335162)
Supplement: S1 Table — Structure of the Generalized Linear Mixed-effects Models adjusted to test the best set of variables to explain the production of reproductive structures of C. minensis cacti associated or not with termite nests. 1. Mean monthly temperature is the mean temperature of the 30 days prior to collection; 2. Mean weekly temperature is the mean temperature of the seven days prior to collection; 3. Monthly rainfall is the accumulated rainfall in the 30 days prior to collection; 4. Weekly rainfall is the accumulated rainfall in the seven days prior to collection; 5 Temperature and substrate interaction are the possible interactions between weekly and monthly temperature variations and the termite nest and rock substrates; 6. Rainfall and substrate interaction are the possible interactions between the weekly and monthly rainfall variations and the termite nest and rock substrates; 7. Substrate are the two substrates to which the cacti are inserted – termite nest and rock substrate. * This formula indicates that possible differences between the cacti in the rock substrate or termite nest were not considered in terms of excess zeros.* The correction for excess zeros was only applied to the fruit and bud models, as it was not necessary for the flower models. (PDF) [file pone.0335162.s002.pdf]

Supporting information

S1 Table. Structure of the Generalized Linear Mixed-effects Models adjusted to test the best set of variables to explain the production of reproductive structures of *C. minensis* cacti associated or not with termite nests. **1 Mean monthly temperature** is the mean temperature of the 30 days prior to collection; **2 Mean weekly temperature** is the mean temperature of the seven days prior to collection; **3 Monthly rainfall** is the accumulated rainfall in the 30 days prior to collection; **4 Weekly rainfall** is the accumulated rainfall in the seven days prior to collection; **5 Temperature and substrate interaction** are the possible interactions between weekly and monthly temperature variations and the termite nest and rock substrates; **6 Rainfall and substrate interaction** are the possible interactions between the weekly and monthly rainfall variations and the termite nest and rock substrates; **7 Substrate** are the two substrates to which the cacti are inserted – termite nest and rock substrate;

* This formula indicates that possible differences between the cacti in the rock substrate or termite nest were not considered in terms of excess zeros.

* The correction for excess zeros was only applied to the fruit and bud models, as it was not necessary for the flowers.

**Models**	**Predictors**							**Covariable**	**Zero inflated**
**Complete**	Mean monthly temperature[1]	Mean weekly temperature [2]	Monthly rainfall[3]	Weekly rainfall[4]	Temperature and substrate interaction[5]	Rainfall and substrate interaction[6]	Substrate[7]		
**Complete without interaction**	Mean monthly temperature	Mean weekly temperature	Monthly rainfall	Weekly rainfall	-	-	Substrate		
**Monthly**	Mean monthly temperature	-	Monthly rainfall	-	Temperature and substrate interaction	Rainfall and substrate interaction	Substrate		
**Monthly without interaction**	Mean weekly temperature	-	Monthly rainfall	-	-	-	Substrate		
**Weekly**	-	Mean weekly temperature	-	Weekly rainfall	Temperature and substrate interaction	Rainfall and substrate interaction	Substrate	Number of stems (Cactus size)	~1*
**Weekly without interaction**	-	Mean weekly temperature	-	Weekly rainfall	-	-	Substrate		
**Climatic monthly**	Mean monthly temperature	-	Monthly rainfall	-	-	-	-		
**Climatic weekly**	-	Mean weekly temperature	-	Weekly rainfall	-	-	-		
**Complete climatic**	Mean monthly temperature	Mean weekly temperature	Monthly rainfall	Weekly rainfall					
**Substrate**	-	-	-	-	-	-	Substrate		
