# Supplementary material for: Increased fruit production in Cipocereus minensis (Cactaceae) associated with termite nests (Isoptera: Termitidae) in Campo Rupestre (Brazilian altitude grassland)
Source: PLoS One. 2025 Nov 14;20(11):e0335162. doi: 10.1371/journal.pone.0335162 (PMC12617911; doi:10.1371/journal.pone.0335162)
Supplement: S2 Table — The AICc and ΔAICc method were used to check which sets were the most parsimonious and best explained the variations. k represents the number of parameters used. (PDF) [file pone.0335162.s003.pdf]

Supporting information

S2 Table. Results of the AICc and ΔAICc method used to check which sets were the most parsimonious and best explained the variations. k represents the number of parameters used.

Models	Flower buds			Flowers			Fruits		
	**k**	**AICc**	**ΔAICc**	**k**	**AICc**	**ΔAICc**	**k**	**AICc**	**ΔAICc**
Complete	**15**	**12885.58**	**0.00**	**13**	**5032.26**	**0.00**	15	9275.48	3.67
Complete without interactions	11	12889.03	3.44	9	5104.84	72.58	11	9328.31	56.50
Monthly	11	12890.06	4.48	9	5054.53	22.26	**11**	**9271.81**	**0.00**
Monthly without interactions	9	12886.06	0.48	7	5103.38	71.11	9	9326.75	54.94
Weekly	11	12890.01	4.42	9	5064.41	32.15	11	9293.11	21.30
Weekly without interactions	9	12887.95	2.37	7	5111.65	79.39	9	9340.07	68.26
Climatic monthly	8	12888.77	3.19	6	5103.99	71.72	8	9330.07	58.26
Climatic weekly	8	12890.69	5.10	6	5112.26	79.99	8	9343.38	71.58
Complete climatic	10	12891.74	6.16	8	5105.45	73.18	10	9331.62	59.82
Substrate	7	12903.24	17.66	5	5124.78	92.52	7	9358.40	86.60
